# Peripapillary choroidal vascularity index and thickness in patients with systemic sclerosis

**DOI:** 10.3389/fmed.2023.1273438

**Published:** 2023-10-16

**Authors:** Barbara Pieklarz, Ewa Gińdzieńska-Sieśkiewicz, Izabela Zawadzka, Magdalena Bagrowska, Joanna Daniluk, Patryk Sidorczuk, Otylia Kowal-Bielecka, Joanna Konopińska, Diana Anna Dmuchowska

**Affiliations:** ^1^Ophthalmology Department, Medical University of Białystok, Białystok, Poland; ^2^Department of Rheumatology and Internal Diseases, Medical University of Białystok, Białystok, Poland

**Keywords:** systemic sclerosis, peripapillary choroidal thickness, glaucomatous optic neuropathy, choroidal vascularity index, retinal nerve fiber layer

## Abstract

**Introduction:**

Patients with systemic sclerosis (SSc) present an increased risk of developing glaucomatous optic neuropathy (GON). We investigated peripapillary choroidal parameters and peripapillary retinal nerve fiber layer (RNFL) thickness using spectral domain optical coherence tomography (SD-OCT) to determine the relationships of these factors with clinical variables.

**Methods:**

A total of 33 patients with SSc were enrolled and compared to 40 controls. After obtaining circular scans around the optic disc, the global and quadrant peripapillary choroidal thickness (pCT) and RNFL thickness were measured. Additionally, the peripapillary choroidal vascularity index (pCVI), which allows for a quantitative analysis of the choroidal vasculature, was determined.

**Results:**

No significant differences were found in pCT and RNFL thickness between patients with SSc and controls, or within SSc subtypes (diffuse cutaneous systemic sclerosis (dcSSc) compared to limited cutaneous systemic sclerosis (lcSSc)) (*p* > 0.05). The pCVI was significantly lower in patients with SSc than in control subjects (64.25 ± 1.94 vs.65.73 ± 2.12, *p* < 0.001).

**Conclusion:**

Our results suggest that the statistically significant decrease in pCVI in patients with SSc compared to the control group is probably due to a decrease in the vascular layer, which would partially explain an increased risk of GON in patients with SSc.

## 1. Introduction

Systemic sclerosis (SSc) is an autoimmune connective tissue disease characterized by chronic progressive tissue and organ fibrosis. Its pathophysiology is complex; however, the process initially involves microvascular damage, followed by an autoimmune response, inflammation, and diffuse fibrosis (1). SSc remains a major medical challenge. Multiple organ-based manifestations are important for its diagnosis and classification. The most common consequences of SSc are digital vasculopathy, gastrointestinal complications, lung fibrosis, pulmonary hypertension, cardiac fibrosis, and renal scleroderma crisis (2).

SSc can be classified into two major disease subsets based on the extent of skin involvement. Cases with proximal skin involvement are classified as diffuse cutaneous systemic sclerosis SSc (dcSSc), whereas cases with skin involvement affecting the limbs distal to the elbows and knees, with or without neck and face involvement, are classified as limited cutaneous systemic sclerosis SSc (lcSSc) (1). Both dcSSc and lcSSc may be associated with internal organ involvement (3).

Many ocular manifestations involving the anterior and posterior segments have also been reported in SSc patients (4–6), and some reports suggest that SSc is a high-risk factor for the development of glaucomatous optic neuropathy (GON), especially normal-tension glaucoma (NTG) (7–11).

The choroid has the highest blood flow in the human body and a high vascular content. The end-arterial nature of the choroidal vasculature and the existence of watershed zones render this layer susceptible to inflammation and ischemia in multisystemic diseases (12). Endothelial cell injury, basement membrane thickening, and pericyte loss in choroidal vessels have been reported in histological studies of SSc patients (13). Macular choroidal thickness has been discussed as a promising inflammatory biomarker in systemic autoimmune diseases, especially those with vascular components (14).

Retinal and choroidal microvascular impairments in SSc patients have been confirmed using fluorescence angiography (FA) (15), optical coherence tomography (OCT) (16–20), and OCT-angiography (OCT-A) (21–25). Most studies on choroidal macular thickness have found that patients with SSc have a significantly thinner macular choroid than healthy subjects, probably due to chronic vascular damage (16–18, 20, 24–26). There are two studies in the literature evaluating CVI in SSc; however, they refer to the macular area and, the results are inconsistent (21, 27). In the peripapillary region, only the vascular density parameters and optic nerve head (ONH) parameters obtained using OCT-A were examined in patients with SSc (17, 19, 23), which is associated mostly with retinal and not choroidal circulation due to the functional and structural heterogeneity of these circulatory systems (24). In recent years, interest in peripapillary choroid thickness has increased. However, the characteristics of the choroid in this area are much poorer than those of the macular area. Studies have revealed thinner peripapillary choroidal thickness not only among patients with NTG and primary open-angle glaucoma (POAG), (23–26), but also in other diseases, such as multiple sclerosis (27), chronic obstructive pulmonary disease (28), high myopia (29), or in patients with vitamin D deficiency (30). In contrary, other studies have shown a thicker peripapillary choroid in some diseases, such as nonarteritic anterior ischemic optic neuropathy (31), Parkinson’s disease (32) and retinal vein occlusions at diagnosis, followed by a decrease at an early follow-up stage (33).

The aim of this study was to investigate peripapillary choroidal parameters and peripapillary retinal nerve fiber layer (RNFL) thickness, and to determine their relationships with clinical variables to gain insight into one of the pathophysiological aspects of SSc. To the best of our knowledge, the present study is the first to analyze peripapillary choroidal thickness together with the peripapillary choroidal vascularity index in patients with SSc, and there are no previous reports on peripapillary choroidal characteristics. With our study we aimed at filling this gap. It is worth noting that choroidal thickness is a rough estimate rather than an accurate marker of choroidal status; hence, we not only determined choroidal thickness, but also calculated the peripapillary choroidal vascularity index (pCVI), a novel OCT-based choroidal quantitative parameter that provides more detailed information about the vascular component of the choroid. We hypothesized that patients with SSc would demonstrate alterations in peripapillary choroidal parameters, which would explain their increased risk of GON.

## 2. Materials and methods

This was a prospective single-center, cross-sectional study conducted between March 2021 and March 2022 at the Ophthalmology Department, Medical University of Bialystok. The protocol of the study was approved by the local Bioethics Committee at the Medical University of Bialystok (decision no APK.002.109.2021) and the study was conducted in accordance with the Declaration of Helsinki. Written informed consent was obtained from each subject before enrolment in the study.

A total of 66 eyes from 33 patients with SSc, diagnosed according to the 2013 ACR/EULAR SSc criteria (28), were enrolled in the study, and the patients were followed up by the Department of Rheumatology and Internal Diseases, Medical University of Bialystok. The control group comprised 80 eyes from 40 ophthalmologically and systemically healthy (self-reported) subjects undergoing routine ophthalmological assessments. The groups did not differ with regard to age, sex, or axial length (AL). All participants underwent ophthalmological examination, including refraction, best corrected visual acuity (BCVA) in Snellen converted into log MAR, slit lamp biomicroscopy, AL measurement (Tomey OA-2000 biometer, Nagoya, Japan), fundus examination, and peripapillary structural spectral domain OCT (SD-OCT, Heidelberg Engineering, Heidelberg, Germany). IOP was measured using a Pascal dynamic contour tonometer (DCT, Zeimer Ophthalmic Systems AG, Port, Switzerland). Blood pressure was measured immediately prior to obtaining the OCT images, after 5 min of rest in a sitting position.

In three eyes, RNFL thinning in the superior, inferior, and inferotemporal quadrants was found with no corresponding scotoma in visual field examination and was associated with a glaucomatous disc appearance (preperimetric glaucoma) (29). The exclusion criteria included: ametropia ≥3 diopters, the presence of fundus pathology, phacoemulsification less than 12 months prior to the examination, history of posterior segment surgery, diabetes, and insufficient quality of OCT images.

Data regarding age, sex, SSc subtype (diffuse and limited), disease duration, autoantibody profile, current smoking status, and systemic treatment were recorded. A history of digital ulcers (present or past), the presence of interstitial lung disease (ILD) confirmed via high-resolution computed tomography (HRCT) of the lungs, and cardiac [elevated N-terminal pro b-type natriuretic peptide (NT-proBNP) or heart fibrosis upon magnetic resonance imaging (MRI)] and joint involvement (arthralgia or joint swelling) were also included in the analysis.

Structural microvascular abnormalities related to the pathophysiological process of SSc were visualized noninvasively using nailfold capillaroscopy (NFC). This safe method is relevant for predicting disease progression and monitoring the effects of treatment (30). NFC was performed using a CapillaryScope 200 Dino-Lite Digital microscope (MEDL4N Pro) and stratified based on the characteristic SSc pattern (capillary density, capillary dimension, abnormal capillary morphology, and presence or absence of hemorrhages), categorized by Cutolo et al. as “early,” “active” and “late” scleroderma patterns (30). Blood parameters including CRP, ESR (after 2 h), and NT-proBNP were measured.

### 2.1. OCT image acquisition and analysis

**Figure 1:**
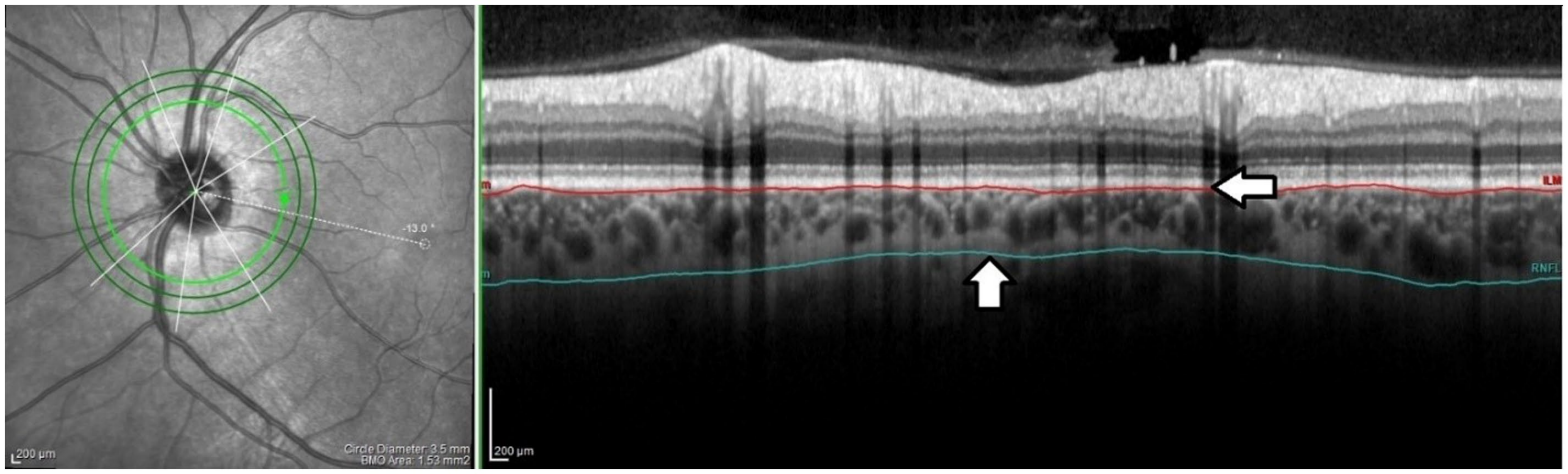
The peripapillary choroidal thickness. The internal limiting membrane (ILM) was manually shifted to Bruch’s membrane (BM) (horizontal arrow) and the RNFL border to the choroidal–scleral junction (CSJ) (vertical arrow). The offset was performed on each scan by the same grader (PS). Peripapillary choroidal thickness was defined as the distance between the BM and the CSJ.

OCT images were taken in mydriasis within the same time interval (12 p.m.–3 p.m.) to avoid diurnal variation in choroidal thickness. Peripapillary OCT images were obtained using a 3.5 mm diameter, 360 degree circle scan centered on the optic nerve head carried out with glaucoma software SD-OCT (Heidelberg Engineering, Heidelberg, Germany). This scan pattern was used to determine the choroidal parameters: peripapillary choroidal thickness (pCT), peripapillary total choroidal area (pTCA), peripapillary luminal area (pLA), peripapillary stromal area (pSA), and retinal nerve fiber layer (RNFL) thickness. RNFL thickness was automatically measured by software and the distribution of the RNFL was displayed as an RNFL thickness map (superior, inferior, nasal, temporal quadrants, and global value). As there is no automatic tool for pCT measurement, pCT was obtained by manually shifting the internal limiting membrane (ILM) to Bruch’s membrane (BM) and the RNFL border to the choroidal–scleral junction (CSJ) (Figure 1). The results were presented as global and quadrant values (superior, inferior, nasal, and temporal) in a thickness map.

**Figure 2:**
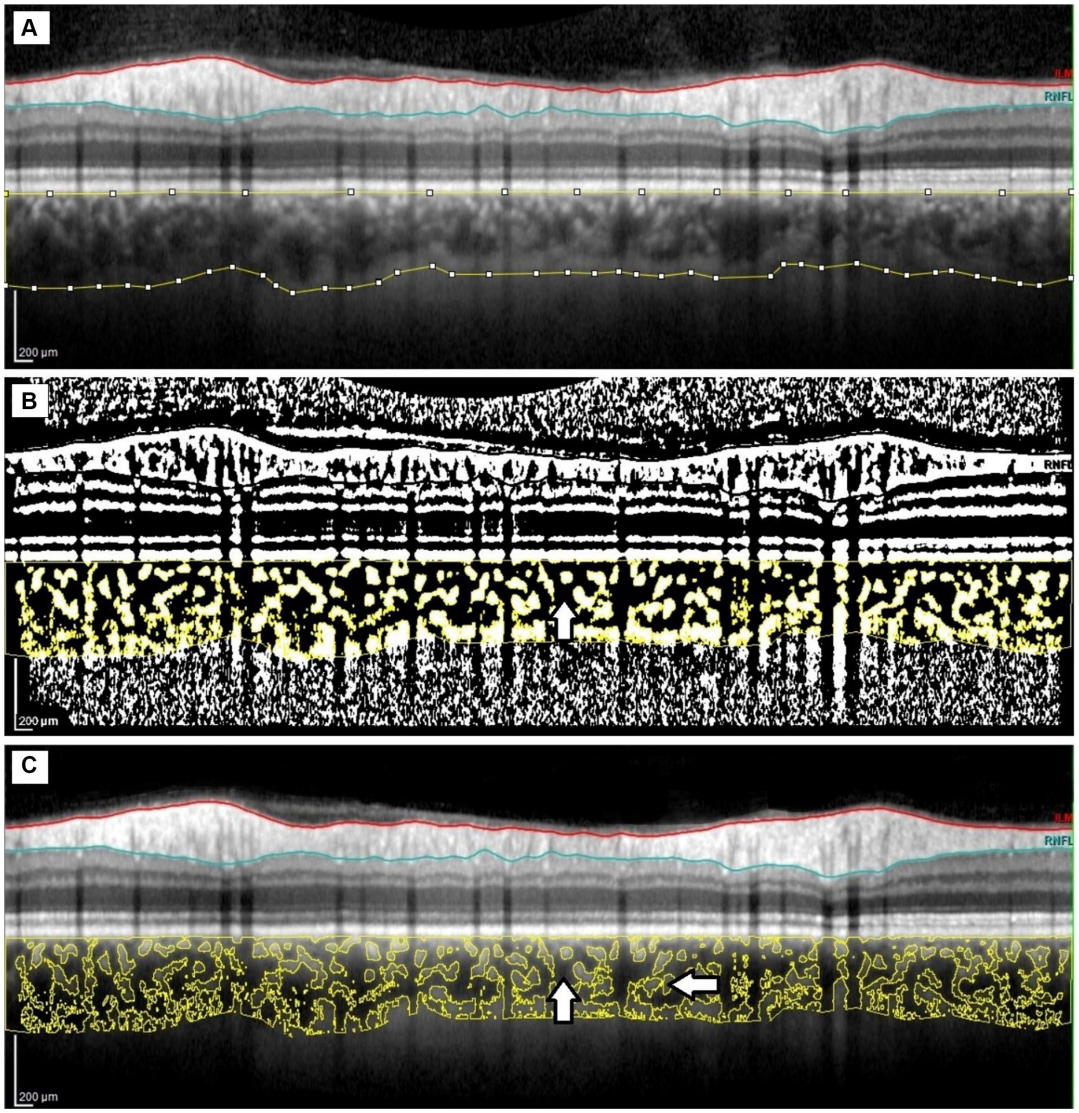
Image binarization of the peripapillary choroid. **(A)** Peripapillary total choroidal area marked on the original OCT circular scan. **(B)** Highlighted luminal area (vertical arrow) using the Color Threshold tool. **(C)**. Overlaying the luminal area on the original OCT scan; luminal area (vertical arrow) and stromal area (horizontal arrow).

Binarization of the peripapillary choroidal area (Figure 2) was performed by two authors (BP and PS). Images were analyzed using the public domain software ImageJ,^1^ using the protocol previously described by Sonoda and Agrawal (31, 33) with a few modifications. The most important modification concerns the setting of the scale, which considered the stretching of the image (OCT sampling density is higher in the axial direction versus the transverse), to avoid erroneous quantification of the measured area (32). An image presented with a 1:1 pixel aspect ratio is stretched axially, but the detailed visualization of the structure is improved compared to a 1 × 1 μm image. Therefore, the scale was set considering the horizontal and vertical scale relationships between the distance and pitch of pixels (μm/pixel) to reflect the actual size of the measured area. A detailed step-by-step image analysis algorithm is provided in the Supplementary material. In the next step, using the Polygon Selection tool, the pTCA was selected from the outer boundary of the RPE–Bruch’s membrane layer to the choroidal–scleral border. The image was converted to an 8-bit image to allow the application of the Niblack Auto Local Threshold tool. The binarized image was reconverted to an RGB image. The vascularized area was highlighted using the Color Threshold tool and pLA and pTCA were measured. pSA was calculated by subtracting pLA from pTCA. The peripapillary choroidal vascularity index (pCVI) was determined as the pLA to pTCA ratio (%).The interobserver reproducibility of the measurements was assessed by measuring the intraclass correlation coefficient (ICC) and absolute agreement. The ICC values for the pCVI, pTCA, and pLA measurements were > 0.85 (95% CI, 0.723–0.986).

### 2.2. Statistical analysis

Analyses were performed using R 4.0.5. statistical software [R Core Team (2021). R: Language and environment for statistical computing by R Foundation for Statistical Computing, Vienna, Austria]. Data are presented as *n* (%) for nominal variables and as mean ± SD or median (Q1; Q3) for continuous variables, depending on the normality of distribution (validated with the Shapiro–Wilk test and based on skewness and kurtosis values). Comparison of groups was made using the chi-square test or the Fisher exact test for nominal data and with the *t* test, Mann–Whitney U test, ANOVA, or Kruskal–Wallis test for continuous variables, as appropriate. *Post hoc* comparisons were based on the Tukey or Dunn test with Bonferroni correction for multiple comparisons. The relationships between continuous variables were assessed using Pearson’s or Spearman’s correlation coefficients, as appropriate. Additionally, linear regression analysis was performed to verify the association between pCVI and demographic, clinical, and ocular features. All calculations were based on *α* = 0.05.

## 3. Results

Sixty-six eyes of 33 SSc patients and 80 eyes of 40 healthy control subjects were enrolled in this study. The groups did not differ with regard to age, sex, axial length, smoking status, or BCVA; however, differences in mean arterial pressure (MAP) and IOP were found. A total of 22 (66.67%) patients presented with dcSSc and 11 (33.33%) had lcSSc. Similarly, there were no significant differences between the two subtypes in terms of age, sex, AL, smoking status, or BCVA, but significant differences existed in MAP and IOP. Detailed demographic and clinical data are reported in Table 1 (SSc group vs. control group) and Table 2 (control group vs. dcSSc vs. lcSSc).

**Table 1:** Demographic and clinical characteristics of SSc patients and control group.

Variable	Control group	SSc group	*p*
Number of patients	40	33	
Number of eyes	80	66	
Age, years, mean ± SD	50.43 ± 10.52	50.97 ± 12.27	0.841^2^
Sex, F, *n* (%)	22 (55.0)	24 (72.7)	0.188
Sex, M, *n* (%)	18 (45.0)	9 (27.3)	
MAP, mean ± SD	97.21 ± 12.43	86.47 ± 9.24	**<0.001** ^**2**^
Nicotine, *n* (%)	6 (15.0)	3 (9.1)	0.484^1^
logMAR, median (Q1;Q3)	0.00 (0.00;0.00)	0.00 (0.00;0.00)	0.686^3^
IOP [mmHg], mean ± SD	15.37 ± 2.13	13.94 ± 3.22	**0.007** ^**2**^
AL, [mm], mean ± SD	23.39 ± 0.97	23.15 ± 0.82	0.104^2^
Duration of the disease [years], median (Q1;Q3)		4.00 (2.00;10.00)	
Pulmonary Involvement, *n* (%)		22 (66.7)	
Cardiac involvement, *n* (%)		11 (33.3)	
Joint involvement, *n* (%)		16 (48.5)	
Digital ulcers (present/history), *n*%		11 (33.3)	
CRP [mg/L], median		1.45 (1.00;3.43)	
ESR [mm/2 h], median		27.00 (18.00;39.00)	
Anti-Scl70 positive, *n* (%)		16 (53.3)	
Anti-centromere positive, *n* (%)		7 (23.3)	
Other Abs positive, *n* (%)		13 (43.3)	
NFC (active/early/late; number of eyes)		30/18/18	

SSc, systemic sclerosis; F, female; M, male; MAP, mean arterial pressure; IOP, intraocular pressure; AL, axial length; CRP, C-reactive protein; ESR, erythrocyte sedimentation rate; h, hours; Abs, antibodies; NFC, nailfold capillaroscopy. Significant differences were tested using chi-square test or Fisher exact test^1^ for nominal variables and with *t*-test^2^ or Mann–Whitney U test^3^ for continuous variables, *p* < 0.05 was considered statistically significant (highlighted with bold).

**Table 2:** Demographic and clinical characteristics of control group and SSc subtypes.

Variable	Control group	dcSSc	lcSSc	*p*	Post-hoc		
					Control group vs. dcSSc	Control group vs. IcSSc	dcSSc vs. IcSSc
Number of patients	40	22	11				
Number of eyes	80	44	22				
Age, years, mean ± SD	50.43 ± 10.52	51.41 ± 13.92	50.09 ± 8.55	0.933^2^			
Sex, F, *n* (%)	22 (55.0)	14 (63.6)	10 (90.9)	0.091^1^			
Sex, M, *n* (%)	18 (45.0)	8 (36.4)	1 (9.1)				
MAP, mean ± SD	97.21 ± 12.43	87.63 ± 10.44	84.13 ± 6.01	**0.001** ^**2**^	**0.009**	**0.005**	0.694
Nicotine, *n* (%)	6 (15.0)	2 (9,1)	1 (9,1)	0.885^1^			
logMAR, median	0.00	0.00	0.00	0.919^3^			
IOP [mmHg], mean ± SD	15.37 ± 2.13	13.58 ± 3.38	14.76 ± 2.75	**0.005** ^2^	**0.003**	0.682	0.294
AL [mm], mean ± SD	23.39 ± 0.97	23.23 ± 0.76	23.00 ± 0.94	0.179^2^			
Duration of the disease [years], median (Q1;Q3)	–	4.00 (2.00;10.00)	5.00 (2.00;10.00)	0.817^3^			
Pulmonary involvement, *n* (%)	–	18 (81.8)	4 (36.4)	**0.018** ^1^			
Cardiac involvement, *n* (%)	–	8 (36.4)	3 (27.3)	0.709^1^			
Joint involvement, *n* (%)	–	8 (36.4)	8 (72.7)	0.071			
Digital ulcers (present/history), *n* (%)	–	9 (40.9)	2 (18.2)	0.259^1^			
Anti-Scl70 positive, *n* (%)	–	16 (80.0)	0 (0.0)	**<0.001** ^1^			
Anti-centromere positive, *n* (%)	–	2 (10.0)	5 (50.0)	**0.026** ^1^			
Other Abs positive, *n* (%)	–	8 (40.0)	5 (50.0)	0.091^1^			

SSc, systemic sclerosis; dcSSc, diffuse SSc; lcSSc, limited SSc; F, female; M, male; BP, blood pressure; IOP, intraocular pressure; AL, axial length; CRP, C-reactive protein; ESR, erythrocyte sedimentation rate; h, hours; Abs, antibodies. Groups compared with chi-square test or Fisher exact test^1^ for nominal variables and with ANOVA^2^ or Kruskal-Wallis test^3^ for continuous variables. *Post hoc* tests used: Tukey test for ANOVA, Dunn test for Kruskal-Wallis test, *p* < 0.05 was considered statistically significant (highlighted with bold).

Table 3 shows the choroidal parameters in detail for the SSc and control eyes. No significant differences were found in the peripapillary choroidal thickness (pCT), RNFL thickness (global and quadrants), peripapillary total choroidal area (pTCA), peripapillary luminal area (pLA), or peripapillary stromal area (pSA) parameters, nor were significant differences found in the aforementioned parameters within the SSc subtype groups (*p* > 0.05 for all). The pCVI was significantly lower in patients with SSc than in healthy control subjects (64.25 ± 1.94 vs. 65.73 ± 2.12, *p* < 0.001), while no significant difference in the pCVI was found between the SSc subgroups, as shown in Table 4.

**Table 3:** Peripapillary choroidal parameters and RNFL thickness comparison between eyes of SSc patients and control group.

Variable	SSc group	Control group	MD	95% CI	*p*
	Mean ± SD	Mean ± SD			
pTCA (μm^2^)	2,403 261.14 ± 689 870.89	2,548 699.87 ± 631 981.03	−145438.73	−367085.20; 76207.75	0.197
pLA (μm^2^)	1,549 602.62 ± 464 458.83	1,678 577.18 ± 430 596.33	−128974.56	−278905.43; 20956.31	0.091
pSA (μm^2^)	853 658.52 ± 231 250.78	870 122.69 ± 210 444.28	−16464.16	−90569.55; 57641.23	0.661
pCVI (%)	64.25 ± 1.94	65.73 ± 2.12	−1.48	−2.15; −0.80	**<0.001**
pCT G (μm)	191.41 ± 59.49	203.05 ± 54.10	−11.64	−30.43; 7.14	0.223
pCT S (μm)	201.26 ± 61.08	218.24 ± 58.52	−16.98	−36.69; 2.73	0.091
pCT I (μm)	174.00 ± 61.13	177.43 ± 55.03	−3.43	−22.65; 15.80	0.725
pCT T (μm)	199.61 ± 70.71	213.36 ± 56.49	−13.76	−35.04; 7.53	0.203
pCT N (μm)	190.91 ± 56.56	203.44 ± 59.21	−12.53	−31.52; 6.46	0.194
pRNFL G (μm)	103.29 ± 10.69	101.33 ± 6.68	1.96	−1.04; 4.96	0.198
pRNFL S (μm)	126.95 ± 16.84	122.29 ± 10.92	4.67	−0.10; 9.43	0.055
pRNFL I (μm)	129.45 ± 18.85	131.25 ± 13.36	−1.8	−7.26; 3.67	0.517
pRNFL T (μm). median (Q1;Q3)	71.00 (65.00;78.00)	69.50 (63.75;75.25)	1.5	−1.00; 5.00	0.255^1^
pRNFL N (μm)	85.38 ± 16.65	82.10 ± 12.59	3.28	−1.64; 8.20	0.19

SSc, systemic sclerosis; pTCA, peripapillary total choroidal area; pLA, peripapillary luminal area; pSA, peripapillary stromal area; pCVI, peripapillary choroidal vascularity index; pCT, peripapillary choroidal thickness; pRNFL, peripapillary retinal nerve fiber layer; G, global; S, superior quadrant; I, inferior quadrant; T, temporal quadrant; N, nasal quadrant; MD, mean difference and CI, confidence interval. Data presented as mean ± SD, unless otherwise indicated. Groups compared with *t*-test or Mann–Whitney U test^1^. *p* < 0.05 was considered statistically significant (highlighted with bold).

**Table 4:** Peripapillary choroidal parameters and RNFL thickness comparison between eyes of control group and SSc group stratified according to subtypes.

					Post-hoc		
Variable	dcSSc group	lcSSc group	Control group	*p*	Control group vs. dcSSc	Control group vs. IcSSc	dcSSc vs. IcSSc
	Mean ± SD	Mean ± SD					
pTCA (μm^2^)	2,425 826.32 ± 726 132.28	2,358 130.79 ± 625 499.99	2,548 699.87 ± 631 981.03	0.397			
pLA (μm^2^)	1,562 003.21 ± 488 305.14	1,524 801.43 ± 423 040.79	1,678 577.18 ± 430 596.33	0.224			
pSA (μm^2^)	863 823.11 ± 244 324.83	833 329.36 ± 206 769.39	870 122.69 ± 210 444.28	0.793			
pCVI (%)	64.13 ± 2.07	64.49 ± 1.67	65.73 ± 2.12	**<0.001**	**<0.001**	**0.038**	0.796
pCT G (μm)	195.89 ± 62.81	182.45 ± 52.45	203.05 ± 54.10	0.311			
pCT S (μm)	206.80 ± 63.57	190.18 ± 55.50	218.24 ± 58.52	0.135			
pCT I (μm)	177.50 ± 67.61	167.00 ± 46.12	177.43 ± 55.03	0.739			
pCT T (μm)	207.77 ± 72.92	183.27 ± 64.56	213.36 ± 56.49	0.143			
pCT N (μm)	191.82 ± 57.18	189.09 ± 56.59	203.44 ± 59.21	0.428			
pRNFL G (μm)	103.73 ± 10.46	102.41 ± 11.35	101.33 ± 6.68	0.343			
pRNFL S (μm)	127.45 ± 19.35	125.95 ± 10.46	122.29 ± 10.92	0.125			
pRNFL I (μm)	127.61 ± 20.72	133.14 ± 14.13	131.25 ± 13.36	0.337			
pRNFL T (μm). median (Q1;Q3)	72.50 (65.75;82.25)	70.00 (64.25;72.75)	70.00 (64.25;77.00)	0.128^1^			
pRNFL N (μm)	85.39 ± 16.15	85.36 ± 18.02	82.10 ± 12.59	0.405			

SSc, systemic sclerosis; systemic sclerosis; dcSSc, diffuse SSc; lcSSc, limited SSc; pTCA, peripapillary total choroidal area; pLA, peripapillary luminal area; pSA, peripapillary stromal area; pCVI, peripapillary choroidal vascularity index; pCT, peripapillary choroidal thickness; pRNFL, peripapillary retinal nerve fiber layer; G, global; S, superior quadrant; I, inferior quadrant; T, temporal quadrant; N, nasal quadrant. Data presented as mean ± SD, unless otherwise indicated. Groups compared with ANOVA or Kruskal-Wallis test^1^. *Post hoc* tests used: Tukey test for ANOVA, Dunn test for Kruskal-Wallis test. *p* < 0.05 was considered statistically significant (highlighted with bold).

No correlation between pRNFL G and pCVI or pRNFL G and pCT G was found between the SSc, dcSSc, or lcSSc groups and the controls, as shown in Supplementary Table S1. The univariate regression analyses of the association between pCVI and demographic, clinical, and ocular features are presented in Table 5 (control group) and Table 6 (SSc group). The univariate regression analysis revealed that in patients with SSc, pCVI was significantly associated with age, pTCA, pLA, global pCT, cardiac involvement, and diuretic use (*p* < 0.05 for all), which was not the case in the control group, for whom only pLA was significantly related to pCVI (*p* = 0.035).

**Table 5:** Regression analysis testing factors associated with pCVI in the control group.

	*β*	SE	*B*	*p*	R^2^	Pseudo R^2^
Age, years	0.013	0.025	0.080	0.624	0.007	−0.020
Sex. male	−0.376	0.533	–	0.486	0.013	−0.013
MAP	−0.010	0.024	−0.079	0.658	0.007	−0.027
Nicotine	0.248	0.741	–	0.740	0.003	−0.024
AL [mm]	−0.034	0.277	−0.020	0.903	0.000	−0.027
pTCA	0.000	0.000	0.246	0.126	0.062	0.037
pLA	0.000	0.000	0.335	**0.035**	0.115	0.091
pSA	0.000	0.000	0.061	0.709	0.004	−0.023
pCVI	–	–	–	–	–	–
pCT Global	0.006	0.005	0.204	0.206	0.043	0.017
pRNFL Global	−0.030	0.041	−0.127	0.436	0.016	−0.010

MAP, mean arterial pressure; AL, axial length, pTCA, peripapillary total choroidal area; pLA, peripapillary luminal area; pSA, peripapillary stromal area; pCVI, peripapillary choroidal vascularity index; pCT, peripapillary choroidal thickness; pRNFL, peripapillary retinal nerve fiber layer; G, global; *β*, beta coefficient; SE, standard error; *B*, standardised beta. Only one eye per patient included into the analysis (eyes with better quality of OCT images selected). *p* < 0.05 highlighted with bold.

**Table 6:** Regression analysis testing factors associated with pCVI in SSc group.

	*β*	SE	*B*	*p*	R^2^	Pseudo R^2^
Age, years	−0.053	0.024	−0.367	**0.026**	0.134	0.106
Sex. male	0.291	0.705	–	0.683	0.005	−0.027
Nicotine	−0.743	1.087	–	0.500	0.015	−0.017
AL [mm]	−0.573	0.380	−0.261	0.142	0.068	0.038
MAP	−0.092	0.033	−0.477	**0.011**	0.208	0.179
pTCA	0.000	0.000	0.395	**0.023**	0.156	0.129
pLA	0.000	0.000	0.464	**0.006**	0.216	0.190
pSA	0.000	0.000	0.252	0.158	0.063	0.033
pCVI	–	–	–	–	–	–
pCT Global	0.012	0.005	0.412	**0.017**	0.169	0.143
pRNFL Global	0.012	0.031	0.072	0.700	0.005	−0.028
dcSSc/lcSSc. IcSSc	0.469	0.663	–	0.484	0.016	−0.016
NFC active = baseline						
Early	−0.384	0.766	–	0.620	0.023	−0.042
Late	−0.629	0.766	–	0.418		
Duration of the disease [years]	0.008	0.043	0.033	0.854	0.001	−0.031
ESR [mm/2 h]	0.008	0.025	0.058	0.759	0.004	−0.033
CRP [mg/L]	0.042	0.155	0.050	0.786	0.003	−0.034
Anti-Scl70 positive	−0.099	0.683	–	0.886	0.001	−0.035
Ant-centromere positive	−0.206	0.805	–	0.800	0.002	−0.033
Other Abs positive	0.515	0.681	–	0.456	0.020	−0.015
Joint involvement	0.337	0.627	–	0.596	0.009	−0.023
Pulmonary involvement	−0.209	0.667	–	0.757	0.003	−0.029
Cardiac involvement	−1.448	0.615	–	**0.025**	0.152	0.124
Digital ulcers (present/history)	0.093	0.668	–	0.890	0.001	−0.032
PDE inhibitors	−0.089	0.629	–	0.888	0.001	−0.032
Ca-blocker	0.767	0.694	–	0.284	0.037	0.006
Hydroxychoroquine	0.093	0.769	–	0.905	0.000	−0.032
Steroids	0.715	0.624	–	0.261	0.041	0.009
Diuretic	−1.320	0.643	–	**0.048**	0.120	0.092

MAP, mean arterial pressure; AL, axial length, pTCA, peripapillary total choroidal area; pLA, peripapillary luminal area; pSA, peripapillary stromal area; pCVI, peripapillary choroidal vascularity index; pCT, peripapillary choroidal thickness; pRNFL, peripapillary retinal nerve fiber layer; G, global; SSc, systemic sclerosis; dcSSc, diffuse SSc; lcSSc, limited SSc, CRP, C-reactive protein; ESR, erythrocyte sedimentation rate; h, hours; Abs, antibodies; PDE, phosphodiesterase; β, coefficient from the regression model; SE, standard error. *p* < 0.05 highlighted with bold. Only one eye per patient included into the analysis (eyes with better quality of OCT images selected).

Additional analysis of choroidal parameters was performed in SSc patients stratified according to SSc pattern using nailfold capillaroscopy. The pTCA, pLA, and pSA values were significantly higher in patients with “late” SSc patterns than those with “active” SSc patterns upon NFC (p < 0.05 for all), and the pCT was significantly thicker (Table 7). The temporal quadrant of pRNFL was significantly thicker in patients with “early” SSc patterns than those with “active” SSc patterns (*p* = 0.009). The groups (“early,” “active,” and “late” SSc patterns on NFC) did not differ with regard to age, sex, AL, MAP, IOP (*p* = 0.589, *p* = 0.484, *p* = 0.509, *p* = 0.07, *p* = 0.648, respectively), or organ involvement (joint, pulmonary, and cardiac involvement; *p* = 0.895, *p* = 0.669, *p* = 0.585, respectively); however, a difference in the presence of digital ulcers (history or present) was found between the “active” SSc pattern group and the “late” SSc pattern group (*p* = 0.003).

**Table 7:** Comparison of peripapillary choroidal parameters and RNFL thickness between eyes stratified according to SSc pattern on nailfold capillaroscopy.

Variable	“Early” SSc pattern	“Active” SSc pattern	“Late” SSc pattern	*p*	*Post hoc*		
					Active vs. Early	Active vs. Late	Early vs. Late
pTCA (μm^2^)	2,316 186.00 ± 707 103.21	2,238 800.32 ± 542 810.82	2,766 335.00 ± 787 284.69	**0.034**	0.921	**0.032**	0.119
pLA (μm^2^)	1,491 862.06 ± 486 531.23	1,445 338.93 ± 371 376.74	1,782 468.12 ± 520 849.43	**0.048**	0.937	**0.046**	0.143
pSA (μm^2^)	824 323.94 ± 226 247.40	793 461.39 ± 177 865.52	983 866.88 ± 272 261.92	**0.020**	0.888	**0.018**	0.090
pCVI (%)	63.99 ± 2.17	64.38 ± 1.94	64.31 ± 1.78	0.796			
pCT G (μm)	181.72 ± 62.76	176.47 ± 44.21	226.00 ± 67.07	**0.012**	0.948	**0.012**	0.055
pCT S (μm)	185.00 ± 64.67	190.27 ± 44.22	235.83 ± 70.64	**0.016**	0.950	**0.028**	**0.029**
pCT I (μm)	171.44 ± 61.40	154.10 ± 44.78	209.72 ± 70.98	**0.007**	0.572	**0.005**	0.121
pCT T (μm)	189.78 ± 76.03	180.93 ± 53.74	240.56 ± 76.91	**0.012**	0.898	**0.011**	0.669
pCT N (μm)	180.94 ± 59.74	180.43 ± 48.94	218.33 ± 59.02	0.052			
pRNFL G (μm)	105.50 ± 9.80	102.03 ± 12.04	103.17 ± 9.28	0.560			
pRNFL S (μm)	128.83 ± 12.94	125.43 ± 21.12	127.61 ± 12.13	0.785			
pRNFL I (μm)	131.61 ± 13.37	129.63 ± 20.04	127.00 ± 21.98	0.768			
pRNFL T (μm), median (Q1;Q3)	74.50 (70.25;79.50)	69.00 (61.25;72.00)	71.50 (68.25;79.00)	**0.046** ^**1**^	**0.009**	0.058	0.237
pRNFL N (μm)	85.89 ± 18.26	85.17 ± 17.57	85.22 ± 14.14	0.989			

SSc, systemic sclerosis; pTCA, peripapillary total choroidal area; pLA, peripapillary luminal area; pSA, peripapillary stromal area; pCVI, peripapillary choroidal vascularity index; pCT, peripapillary choroidal thickness; pRNFL, peripapillary retinal nerve fiber layer; G, global; S, superior quadrant; I, inferior quadrant; T, temporal quadrant; N, nasal quadrant. Data presented as mean ± SD, unless otherwise indicated Groups compared with ANOVA or Kruskal-Wallis test^1^. *Post hoc* tests used: Tukey test for ANOVA, Dunn test for Kruskal-Wallis test. *p* < 0.05 was considered statistically significant (highlighted with bold).

## 4. Discussion

With mounting clinical evidence indicating the involvement of the peripapillary choroid in glaucoma (34–38), it has become increasingly important to detect changes in the choroid in patients with a high risk of developing of glaucomatous changes. As we mentioned above, there are no previous reports on peripapillary choroidal thickness in patients with SSc. Several pathophysiological pathways that might be involved in normal-tension GON have been discussed (39). As the blood supply of the prelaminar region partly derives from branches within the peripapillary choroid, the choroid has been implicated in the pathogenesis of GON and many studies have investigated this relationship (40–43). We not only investigated peripapillary choroidal thickness, but also determined the peripapillary choroidal vascularity index (pCVI) to characterize the choroid in detail in SSc patients. This index reflects the vascular content of the choroid. The CVI provides more detailed information about the vascular component of the choroid across all layers, including the choriocapillaris, Sattler’s layer, and Haller’s layer. The current literature suggests that the CVI has less variability and is influenced by fewer physiological factors than choroidal thickness; therefore, it can be considered a relatively stable parameter for evaluating changes in choroidal vasculature in several chorioretinal and optic disc diseases, including glaucoma (44). The CVI has been proposed as a potential biomarker for establishing early diagnosis, monitoring disease progression, and prognosticating for these patients (45–47). As other authors have emphasized, CVI should be viewed not as an isolated marker, but as an addition to existing parameters such as CT. Specific data on the choroidal stromal and vascular area should also be analyzed (44). In our study, the pCVI was significantly lower in patients with SSc than in healthy control subjects, and no significant difference in pCVI was found between the SSc subtypes. Interestingly, no significant difference was found in pTCA, pLA, and pSA parameters between the SSc and control groups, nor between the SSc subtypes. However, the mean pTCA and pLA values were lower in the SSc group than in the control group, as were the global and quadrant pCT, but the differences were not statistically significant. No correlations were found between pCT, pCVI, and pRNFL within the two groups. The pSA values were comparable between the SSc and control groups. These differences were less pronounced in the SSc subtype groups. Increases or decreases in CVI may be due to various mechanisms; for example, an increase in CVI could be caused by an increase in the number or diameter of the vascular channels in the choroid. In turn, a decrease in CVI could be the result of attenuation of the choriocapillaris, reduction in choroidal vessel size, or loss of large choroidal vessels (45). Our results suggest that the statistically significant decrease in pCVI in patients with SSc compared to the control group is probably due to a decrease in the vascular layer within the peripapillary area. A thinner choroid (global and quadrant) may support this hypothesis, although the differences were not statistically significant. Choroidal thickness is mainly determined by the thickness of Sattler’s and Haller’s layers (25). However, it is possible that changes in the vascular components of the peripapillary choroid in SSc patients may mainly result from damage to the choriocapillary layer, where the vessel lumen is smallest. There are two studies evaluating CVI in SSc, and the results are inconsistent; some authors found no significant differences (21), while others showed reduced CVI values, especially in the dSSc group, compared to the control group (27). However, they refer to the macular area. These results should not be directly compared with ours because they analysed a different area. The anatomy (the end-arterial nature of the choroidal vasculature with strictly segmental blood flow and the existence of watershed zones) and function of the choroid are very complex (12).

There is a discrepancy in the literature regarding the association between age and CVI. Agrawal et al. compared the factors affecting SFCT and CVI in healthy subjects and concluded that only SFCT was affected by age (48). In contrast, Kocak et al. demonstrated decreased LA, TCA, and CVI in healthy eyes with increasing age, with no significant differences in SA. The differences were more significant in the group 0–10 years old (49). The above studies refer to the different studied widths of the choroid in the macula. Guduru et al. stated that in healthy subjects, peripapillary CVI significantly increases after the age of 45 years, which introduces even greater ambiguities in the assessment and interpretation of the relationship between CVI and age, especially in the peripapillary area (50). In our study, the regression analysis revealed that in patients with SSc, pCVI was significantly associated with age, but this was not the case in the controls. Through regression analyses of the control group, we were able to identify pLA as a factor related to pCVI. Interestingly, in the SSc group, some variables, such as age, MAP, cardiac involvement, and diuretic use, were related to pCVI values. Consequently, CVI seemed to be more dependent on various factors in patients with SSc than in the controls.

No significant difference was found in pCT (global and quadrants) between the SSc and control groups, nor within the SSc subtypes. This is in contrast to previous reports that found thinner macular choroids in patients with SSc (16–18, 20, 24–26).

Significant differences were found between controls and patients with SSc in terms of MAP and IOP; however, the choroid shows some autoregulatory capacity during changes in ocular perfusion pressure, which depends on diastolic and systolic blood pressure and IOP (51). In another study, no associations were found between pCT and either MAP or IOP in healthy subjects (52) which is consistent with our analysis (Supplementary Table S2). The control group was comprised of ophthalmologically and systemically healthy patients; therefore, these factors seemed to have no impact on the final results.

The SSc and control groups differed significantly with regard to IOP, and further analyses showed that the dcSSc group differed in IOP compared to the control group. IOP was similar within the SSc subtypes and between eyes stratified according to SSc pattern using nailfold capillaroscopy. It seems that this difference did not affect the thickness of the peripapillary choroid. In a study by Huang et al., the average pCT in healthy controls decreased linearly with age, but other factors, such as IOP or MAP, were significantly related to average pCT (52). On the other hand, as patients with dcSSc presented with lower IOP values, one may speculate that this could have a protective effect, compensating for thinner choroids in the context of GON pathogenesis.

The thinning of RNFL as a result of progressive loss of ganglion cell axons is a cardinal feature of GON (53). Few studies have evaluated the structural glaucomatous abnormalities detected by OCT in patients with SSc; those that exist show conflicting results, but indicate that the detected abnormalities suggest SSc to be a major risk factor for the development of glaucomatous changes. Based on swept-source OCT (SS-OCT), Agapito Tito et al. observed a significant decrease in RNFL temporal quadrant thickness in patients with SSc compared to controls, but no differences were found in RNFL thickness and the macular ganglion cell complex (GCC) between the SSc subtypes. There was also an inverse correlation between disease duration and RNFL thickness and GCC. No significant correlations were found between NFC and OCT parameters. However, patients with osteoarthritis were included in the control group (7). In another study, the authors observed a thinner RNFL in the lower quadrant (SD-OCT) only in patients with an excavation/vertical disc ratio of 0.5 when compared with the control group (8). Hekimsoy et al. investigated OCT-A parameters of ONH and RNFL thickness using SD-OCT in patients with lcSSc. No significant differences were found in peripapillary vessel density and RNFL thickness in lcSSc patients compared with controls (54). This finding is consistent with our results, although dcSSc patients were not enrolled in the above-mentioned study. Our study group was divided into SSc subtypes; no significant difference was found in RNFL thickness between the lcSSc and dcSSc groups and the control group. However, when groups were categorized according to NFC, the temporal quadrant of pRNFL was significantly thicker in patients with an “early” SSc pattern than in those with an “active” pattern. Gomes et al. evaluated the association between capillaroscopy patterns and the presence of glaucoma in patients with SSc, but found no significant differences, although the SSc patterns in that study were classified as mild or severe (11). However, the basis of the glaucoma diagnosis was not specified and the NFC classification differed from our study design. The mechanisms underlying the reduction in temporal RNFL thickness in SSc have not been entirely elucidated, but could be associated with greater susceptibility to vasoconstriction, endothelial damage, or ischemia in this region (7). Due to inconclusive data, further investigations focusing on the relationship between GON and SSc are required to address this issue.

The presence of giant capillaries is characteristic of “early” and “active” scleroderma patterns, while the presence of severely lowered density combined with abnormal shape is typical of “late” scleroderma patterns (30). The loss of capillaries, vascular architectural disorganization, and the presence of ramified/bushy capillaries (“late” SSc pattern) represents the clearest aspect of advanced SSc microvascular damage, regardless of the presence of a limited or diffuse subtype (55).

Sub-analysis of scleroderma patients stratified according to NFC pattern showed interesting results. Statistically significant differences in pTCA, pLA, pSA, and pCT were observed. The higher values for TCA, LA, and SA, and a thicker peripapillary choroid (except for the nasal quadrant) in patients with a “late” capillaroscopy SSc pattern compared to those with an “active” SSc pattern were surprising. No significant difference in pCVI was found between “early,” “active,” and “late” SSc pattern groups. Due to the small number of patients in each group, this issue requires further investigation. Shenavandeh et al. assessed retinal vascular changes in SSc patients, but found no evidence of a relationship between retinal vascular changes seen on fundus photography and SSc patterns upon NFC (56). However, this cannot be related to our study due to the functional and structural heterogeneity of retinal and choroidal circulation (57, 58).

There is a discrepancy between peripapillary choroidal thickness found in our study and studies by Fard et al. (59) and Huang et al. (52). Choroidal thickness measurements can vary significantly using three different definitions of the choroidal-scleral junction as posterior boundaries (60). In our study, choroidal thickness measurements were based on the identification of the outer border of the choroid stroma (Figure 1) which was also clearly seen after binarization of the images (Figure 2). Vuong et al. showed that this measurement method was more reproducible than the other methods (60).

The limitations of the present study, which must be taken into account when interpreting the results, are the relatively small number of patients due to the rarity of the disease. A technical limitation is the possibility of erroneously high CVI measurements due to shadowing of the large superficial retinal vessels on circle peripapillary OCT scans, but this would apply to all studied subjects. The study was conducted during the COVID-19 pandemic, causing the study period to be shortened, as scheduled hospital admissions were reduced. Additionally, except for three patients with SSc and preperimetric glaucoma, no patients with both SSc and more advanced glaucoma were included in the study. Such a group could potentially present more severe damage of the choroid. Although our study provides insight into the pathophysiology of the choroid in patients with systemic sclerosis, longitudinal observation would facilitate future studies by documenting pRNFL changes over time and the possible development of GON in patients with SSc. Inclusion of not only RNFL, but also ganglion cell complexes [as a more sensitive parameter for risk/occurrence of glaucoma (61)] in the analysis could also be informative.

In conclusion, our cohort of patients with SSc presented alterations of choroidal characteristics. The differences were variable depending on the approach to patient stratification. A decrease in pCVI in patients with SSc compared to the control group reflects a decrease in the vascular layer within the peripapillary area, which would support the vascular hypothesis for an increased risk of GON in patients with SSc.

## Data availability statement

The original contributions presented in the study are included in the article/Supplementary material, further inquiries can be directed to the corresponding authors.

## Ethics statement

The studies involving humans were approved by Medical University of Białystok. The studies were conducted in accordance with the local legislation and institutional requirements. The participants provided their written informed consent to participate in this study.

## Author contributions

BP: Conceptualization, Data curation, Formal analysis, Investigation, Methodology, Visualization, Writing – original draft. EG-S: Data curation, Investigation, Supervision, Writing – review & editing. IZ: Investigation, Writing – original draft. MB: Investigation, Writing – original draft. JD: Investigation, Writing – original draft. PS: Investigation, Methodology, Writing – original draft. OK-B: Project administration, Supervision, Writing – review & editing. JK: Project administration, Supervision, Writing – review & editing. DD: Conceptualization, Formal analysis, Funding acquisition, Investigation, Methodology, Project administration, Supervision, Writing – review & editing.

## Footnotes

^1^ https://imagej.nih.gov/ij/
