# Supplementary material for: Peripapillary choroidal vascularity index and thickness in patients with systemic sclerosis
Source: Front Med (Lausanne). 2023 Oct 16;10:1273438. doi: 10.3389/fmed.2023.1273438 (PMC10617027; doi:10.3389/fmed.2023.1273438)
Supplement: Supplementary file 1 [file Table_1.docx]

**
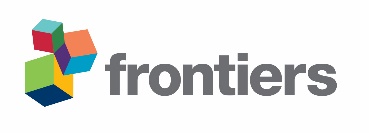
**

***Supplementary Tables***

| **Table S1**. Correlations between peripapillary RNFL thickness with both peripapillary choroidal vascular index and peripapillary choroidal thickness values in controls and patients with SSc | | | | | | | | | | | |
| --- | --- | --- | --- | --- | --- | --- | --- | --- | --- | --- | --- |
| **Characteristic** | | | **Control group** | | **SSc** | | **dcSSc** | | **lcSSc** | | |
|  |  |  | **rho** | **p** | **rho** | **p** | **rho** | **p** | **rho** | **p** |  |
| pRNFL G | vs. | pCVI | -0.14 | 0.373 | 0.09 | 0.608 | 0.25 | 0.265 | -0.52 | 0.107 |  |
| pRNFL G | vs. | pCT G | 0.09 | 0.563 | -0.09 | 0.629 | 0.04 | 0.866 | -0.42 | 0.203 |  |
| Abbreviations: SSc, systemic sclerosis; dcSSc, diffuse SSc; lcSSc, limited SSc; pRNFL G, peripapillary retinal nerve fiber layer global; pCVI, peripapillary choroidal vascularity index; , pCT G, peripapillary choroidal thickness global.  *rho - Pearson's or Spearman's correlation coefficient; Only one eye per patient included into the analysis (eyes with better quality of OCT images selected).* | | | | | | | | | | |  |

| **Table S2**. Correlations between intraocular pressure and mean arterial pressure with peripapillary choroidal thickness in controls and SSc patients. | | | | | | |
| --- | --- | --- | --- | --- | --- | --- |
|  |  |  | **Control group** | | **SSc group** | |
| **Characteristic** | | | **rho** | **p** | **rho** | **p** |
| IOP | vs. | pCT G | 0,02 | 0,894 | 0,04 | 0,836 |
| IOP | vs. | pCT S | -0,06 | 0,733 | 0,01 | 0,961 |
| IOP | vs. | pCT I | 0,08 | 0,634 | 0,12 | 0,568 |
| IOP | vs. | pCT T | -0,02 | 0,924 | -0,01 | 0,976 |
| IOP | vs. | pCT N | 0,13 | 0,442 | 0,04 | 0,836 |
| MAP | vs. | pCT G | -0,24 | 0,300 | -0,17 | 0,556 |
| MAP | vs. | pCT S | -0,21 | 0,366 | -0,30 | 0,292 |
| MAP | vs. | pCT I | -0,43 | 0,061 | -0,02 | 0,940 |
| MAP | vs. | pCT T | 0,11 | 0,636 | -0,19 | 0,518 |
| MAP | vs. | pCT N | -0,31 | 0,188 | -0,12 | 0,684 |
| Abbreviations: SSc, systemic sclerosis; IOP, intraocular pressure; MAP, mean arterial pressure; pCT, peripapillary choroidal thickness,G, global; S, superior quadrant; I, inferior quadrant; T, temporal quadrant; N, nasal quadrant.  *rho - Pearson's or Spearman's correlation coefficient; Only one eye per patient included into the analysis (eyes with better quality of OCT images selected).* | | | | | | |
