# Supplementary material for: Peripapillary choroidal vascularity index and thickness in patients with systemic sclerosis
Source: Front Med (Lausanne). 2023 Oct 16;10:1273438. doi: 10.3389/fmed.2023.1273438 (PMC10617027; doi:10.3389/fmed.2023.1273438)
Supplement: Supplementary file 2 [file Data_Sheet_1.PDF]

Supplementary Material

STEP-BY-STEP binarization of images and choroidal vascularity index (CVI) calculations

1. The 1 x 1 pixel image of the peripapillary OCT scan (3.5 mm diameter, 360 degree circle scan centered on the optic nerve) (Heidelberg Engineering, Heidelberg, Germany) was opened in ImageJ software (https://imagej.nih.gov/ij, version 1.53k, U.S. National Institutes of Health, Bethesda, MD, USA).
2. The scale was set as follows; the image was enlarged and the line tool was used to measure a pixel length of 200 µm, as given in the horizontal scale at the bottom of the OCT scan.
The known horizontal distance (yellow frame) was entered into the scale-set window. The horizontal scale was automatically calculated (red frame).

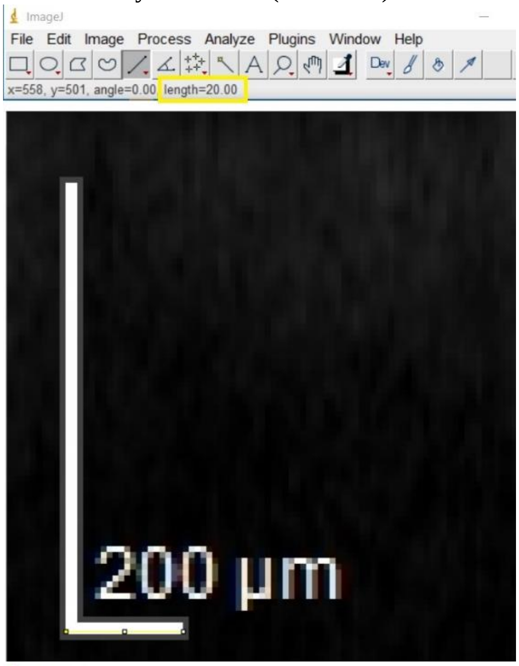

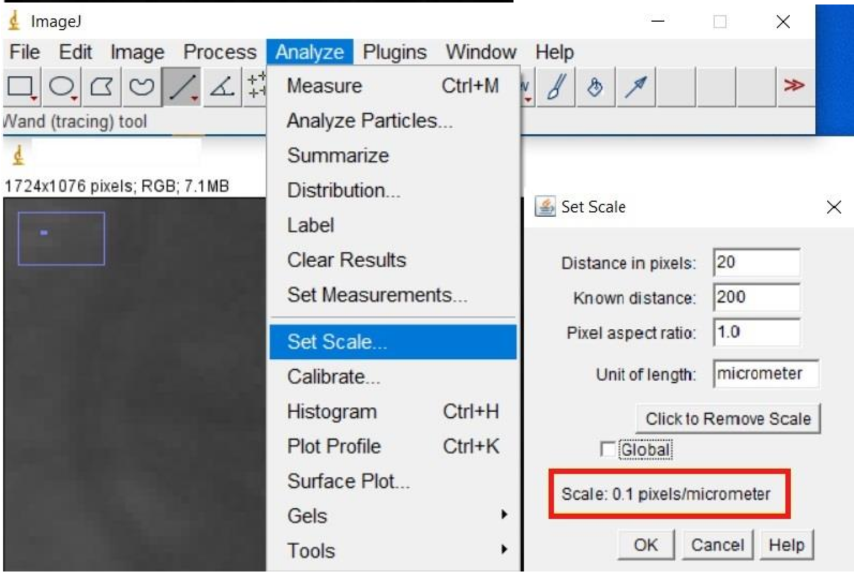

The scale was reset (green frame) and the vertical scale was calculated similarly.

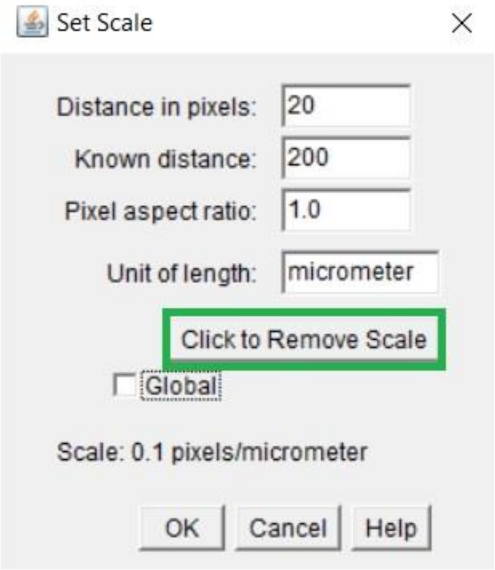

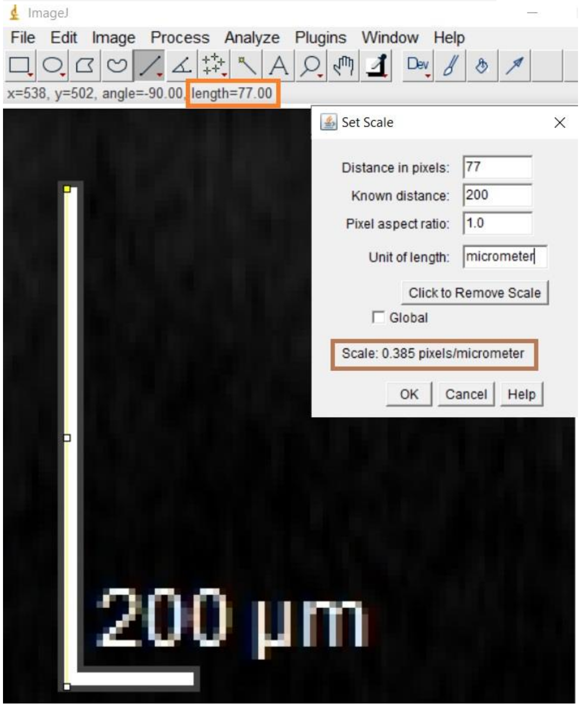

The pixel aspect ratio was calculated by dividing the horizontal pixel values by the vertical pixel values (20/77=0.25974026).

Once again, the horizontal scale was entered and the rounded result of the pixel aspect ratio (0.26) was entered into the pixel aspect ratio window. The unit of length was micrometers. For further image analyses, the “global” option was checked.

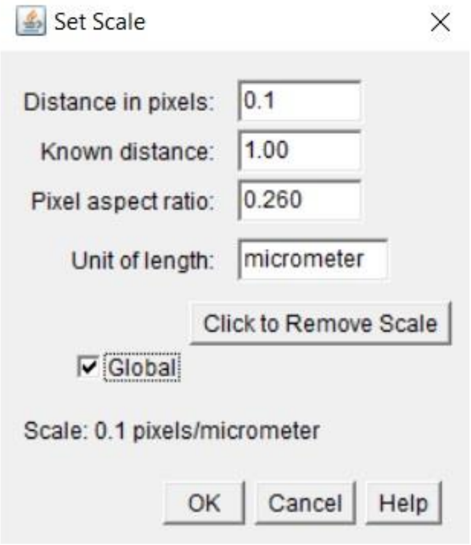

3. The Polygon tool (yellow frame) was used to select the area between the outer boundary of the RPE–Bruch's membrane layer and the choroidal–scleral junction.

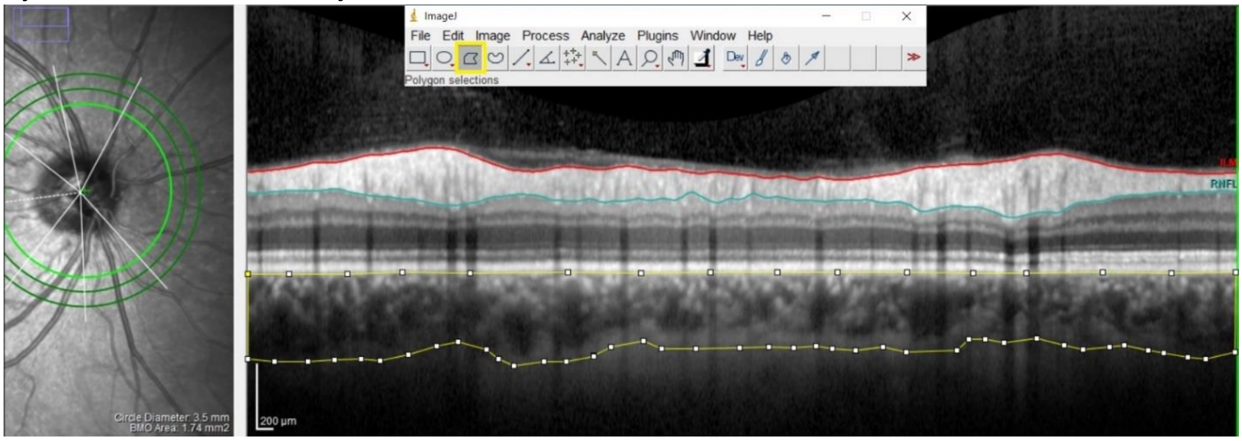

4. The polygon area representing the region of interest (ROI) was added to the ROI manager (by clicking the left mouse button on this area).

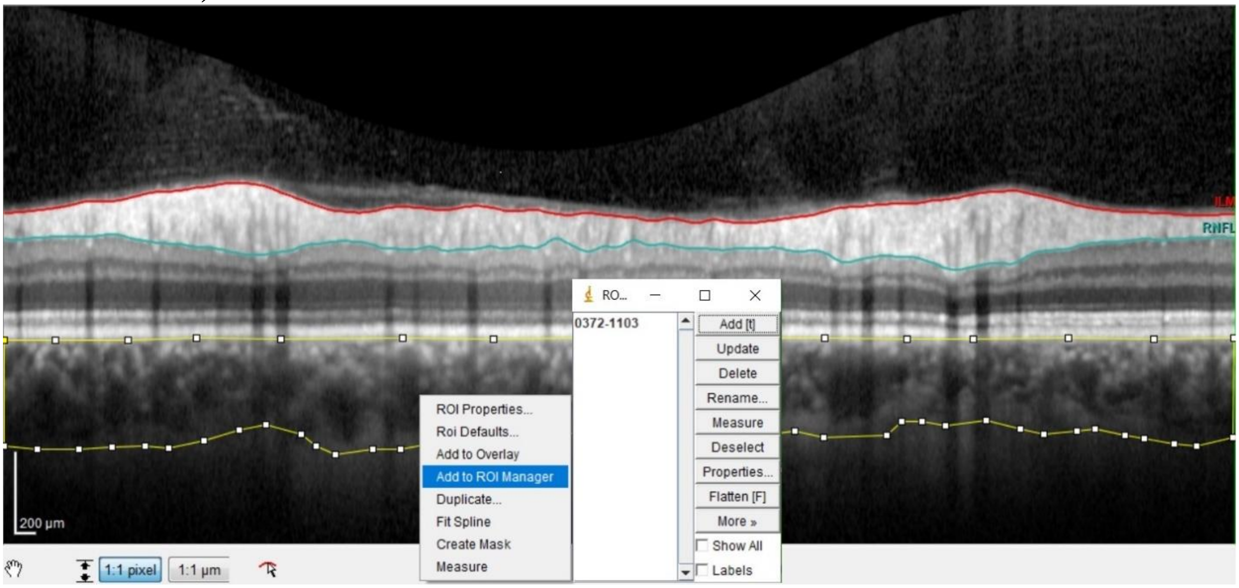

5. The image was converted to an 8-bit image.

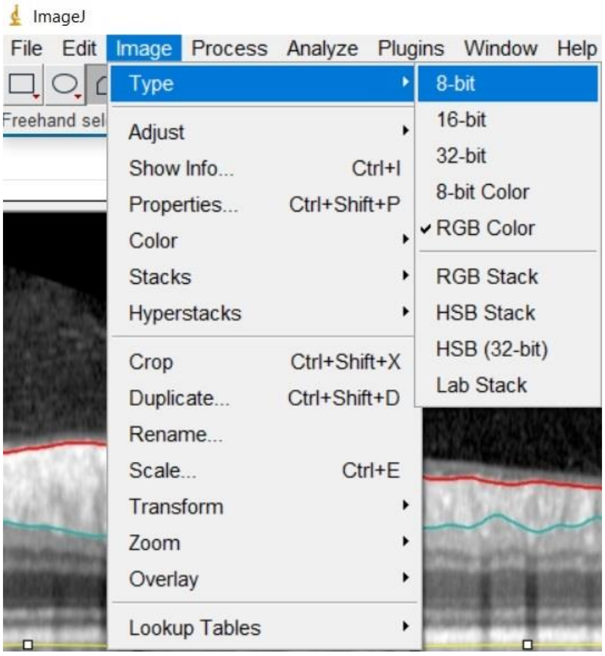

6. The image was adjusted to Niblack with the Auto Local Threshold tool.

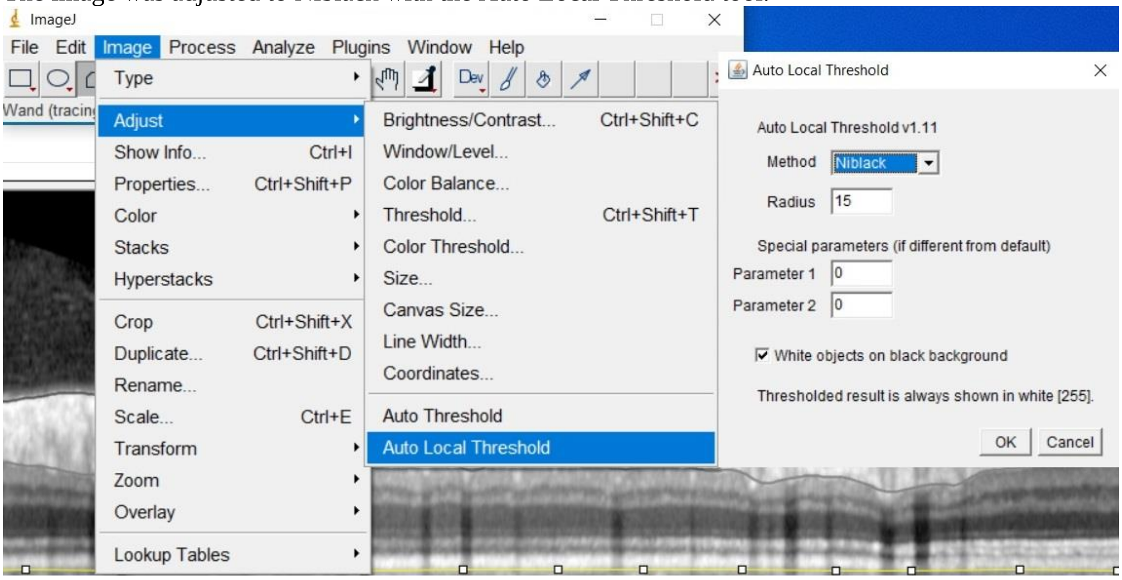

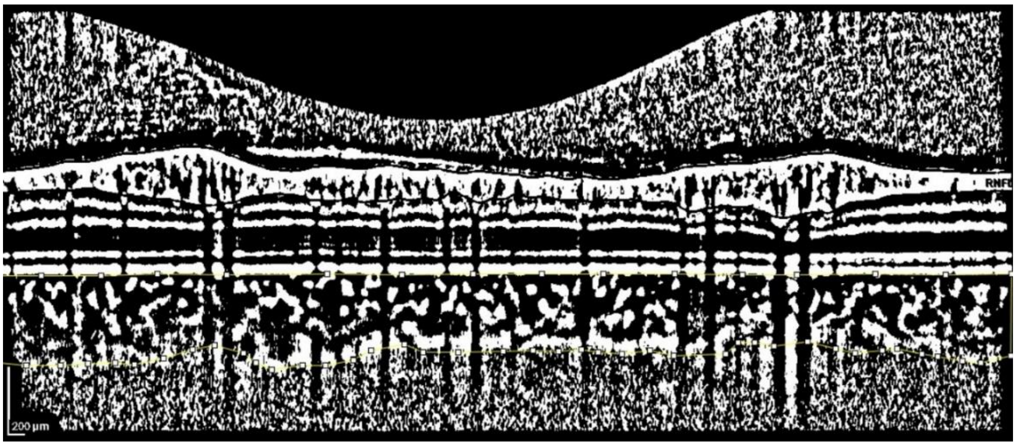

7. The binarized image was reconverted to an RGB image.

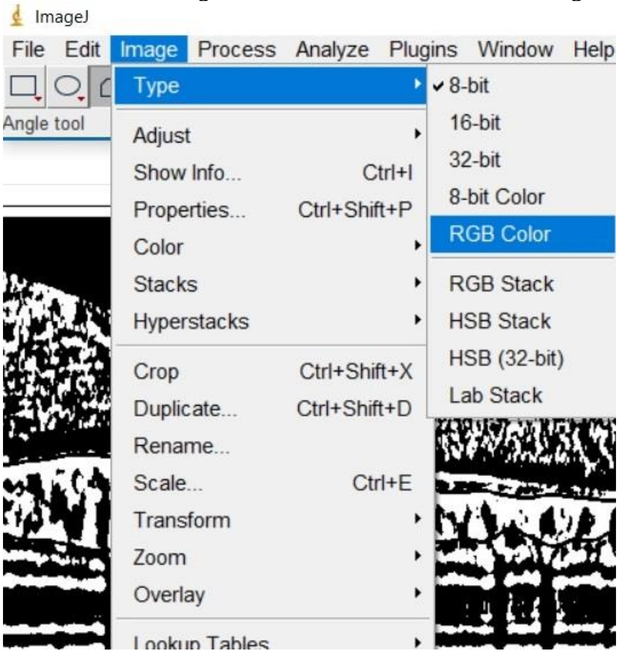

8. The area of vascularity was highlighted by selecting the Color Threshold tool. The first bar under brightness was adjusted to 0 (orange frame) and the second to 254 (blue frame). This was confirmed by clicking SELECT.

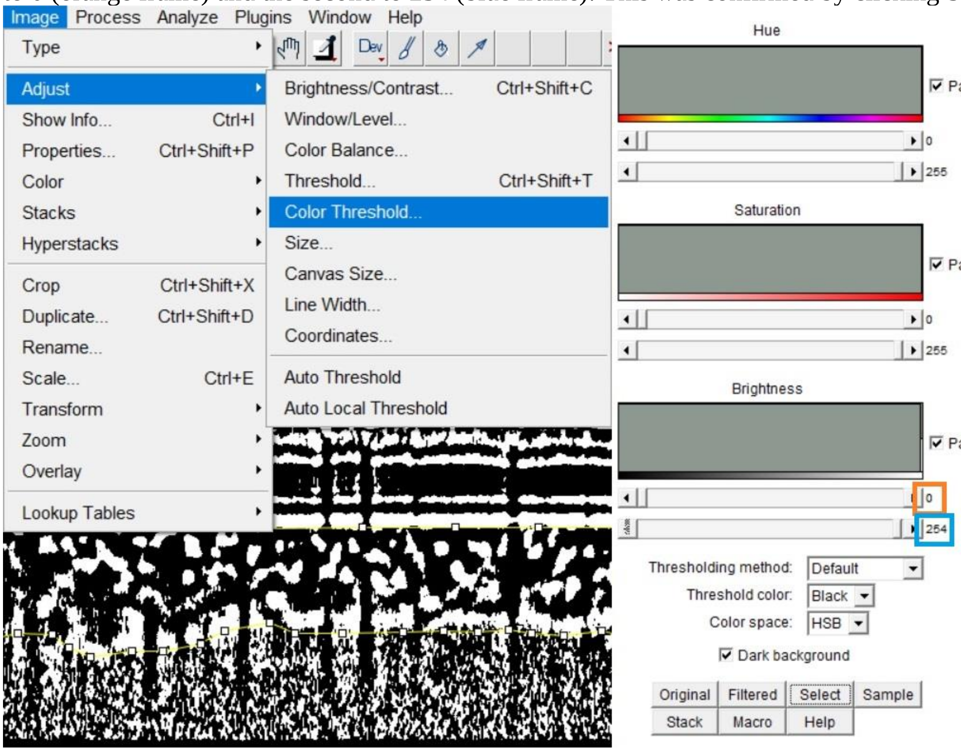

9. The highlighted area was added to the ROI manager (by clicking the left mouse button on this area).

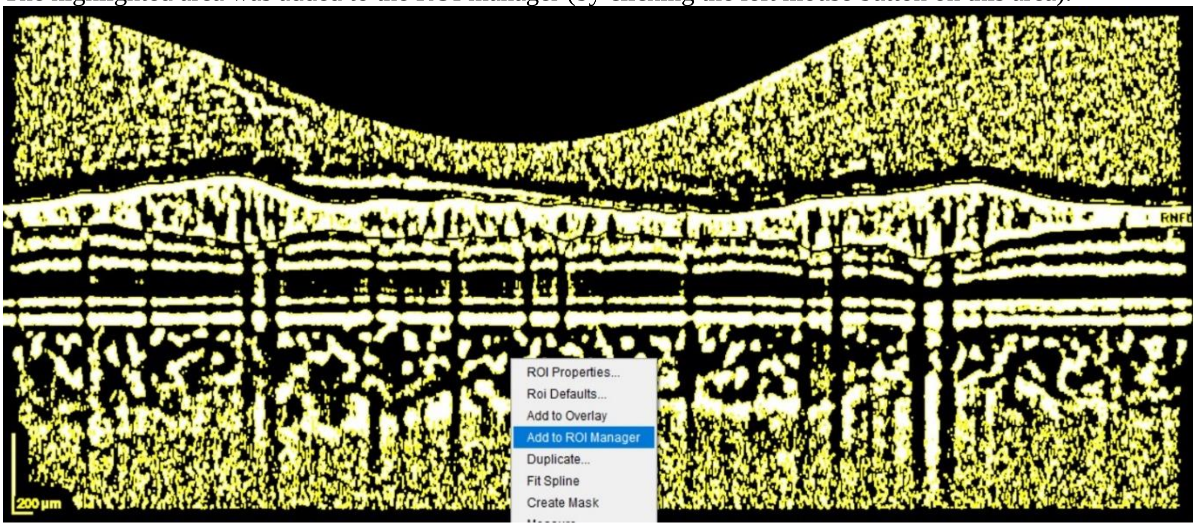

10. Both areas in the ROI manager were selected by holding the CTRL button on the keyboard and merged by selecting More < AND. In the next step, the highlighted area within the selected polygon was added to the ROI manager (by clicking the left mouse button in this area).

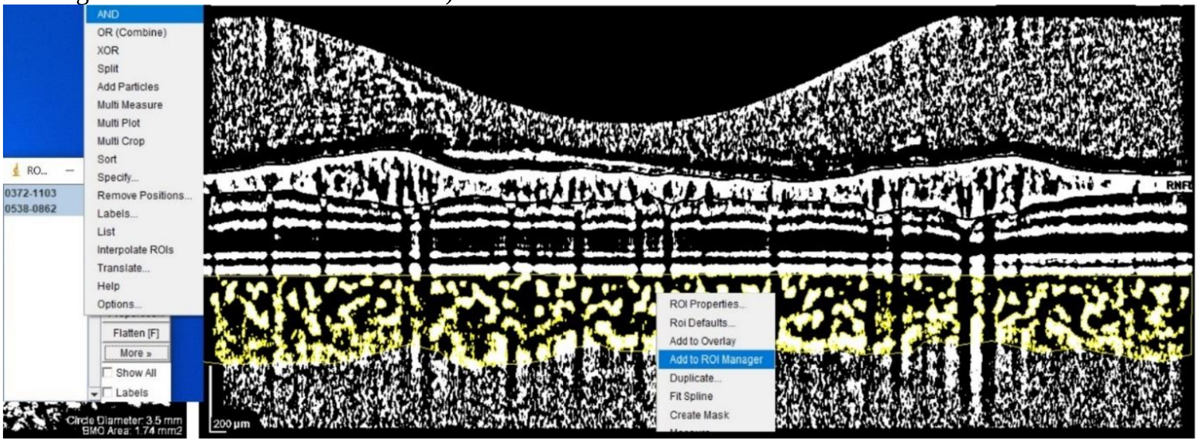

11. Lastly, the first area (green frame), corresponding to the peripapillary total choroidal area (pTCA), and the third area (yellow frame), which represents the peripapillary luminal area (pLA), were measured in the ROI manager by clicking the button MEASURE. The measured areas are displayed in square micrometers.

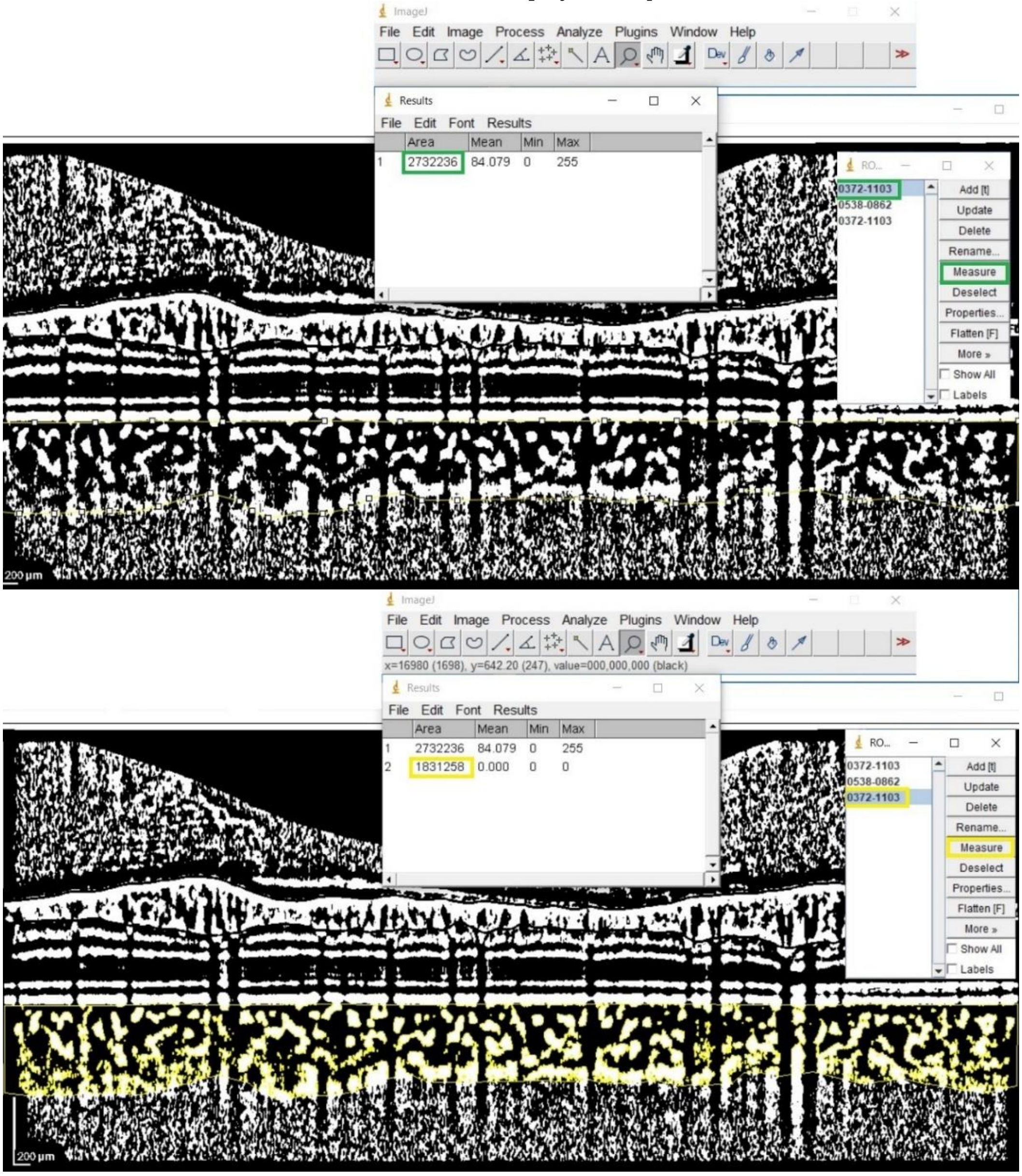

12. The peripapillary stromal area (pSA) was obtained after subtraction of the pLA from the pTCA (2732236-1831258=900978 μm^2).

13. The peripapillary choroidal vascularity index (pCVI) was calculated as (pLA/pTCA)x100%: (1831258:2732236)x100%=67.02%.
